# Transplantable programmed death ligand 1 expressing gastroids from gastric cancer prone *Nfkb1*^*−/−*^ mice

**DOI:** 10.1038/s41419-021-04376-2

**Published:** 2021-11-17

**Authors:** Jun T. Low, Gwo-Yaw Ho, Mark Scott, Chin Wee Tan, Lachlan Whitehead, Kathy Barber, Hon Y. K. Yip, Johanna F. Dekkers, Yumiko Hirokawa, John Silke, Antony W. Burgess, Andreas Strasser, Tracy L. Putoczki, Lorraine A. O’Reilly

**Affiliations:** 1 The Walter and Eliza Hall Institute of Medical Research, Parkville, VIC Australia; 2 Department of Medical Biology, The University of Melbourne, Parkville, VIC Australia; 3 School of Clinical Sciences, Monash University, Clayton, VIC Australia; 4 Princess Máxima Center for Pediatric Oncology, Utrecht, CT The Netherlands; 5 Department of Surgery, The University of Melbourne, Parkville, VIC Australia; 6 Present Address: Translational Research Institute Australia, Woolloongabba, QLD Australia; 7 Present Address: Monash Biomedicine Discovery Institute and Department of Biochemistry and Molecular Biology, Monash University, Clayton, VIC Australia; 8 Present Address: Cancer Genomics Netherlands, Oncode Institute, Utrecht, CG The Netherlands

**Keywords:** Cancer models, Gastric cancer

Gastric cancer (GC) is the fifth most common cancer and the third highest cause of cancer-related deaths globally [1]. There are several histological subtypes of GC [2], with intestinal type GC (IGC) the most common. IGC is initiated by inflammatory gastritis, often driven by *Helicobacter pylori (H. pylori)* or EBV infection [1]. This results in sustained activation of NF-κB transcription factors, which drive the expression of inflammatory factors thought to promote tumorigenesis [2]. Programmed death ligand 1 (PD-L1) is the ligand of the immune checkpoint regulator PD-1 that is expressed on T cells. The PD-L1/PD1 interaction inhibits cytotoxic T cell mediated killing of cancer cells [3]. Tumours can hijack this pathway, for example by expressing PD-L1, to render them resistant to such immune attack [3]. Immune checkpoint inhibitor therapy (ICIT) enhances the killing of malignant cells, including GC [4], by blocking PD1 or CTLA4 or the PD1 ligands, PDL1/PDL2.

We developed a mouse model of IGC that is driven by loss of NF-κB1, a member of the REL/NF-κB family of transcription factors. *Nfkb1*^*−/−*^ mice present with abnormally increased expression of TNF and activation of STAT1 in the stomach, resulting in an inflammatory immune response that culminates in the development of GC [5, 6]. Cells within the stomachs from pre-neoplastic *Nfkb1*^*−/−*^ mice display abnormally increased proliferation, JAK/STAT1 signalling and PD-L1 expression [5, 6] (Supplementary Fig. 1A**)**. Pertinently, polymorphisms in human *NFKB1* that diminish its function have been linked with increased risk for GC [6]. The EBV^+^ and Microsatellite Instability^hi^ (MSI^hi^) subtypes of human GC [7] exhibit features similar to the GC that arise in *Nfkb1*^*−/−*^ mice, suggesting that they are also driven by sustained inflammation, immune activation and that they may benefit from ICIT [6]. Organoids derived from human GC tissue and from animal models are an important experimental tool in GC research and for the testing of novel targeted therapies [8].

We generated gastric organoids (GOs) from the stomachs of both young, healthy and older tumour bearing *Nfkb1*^*−/−*^ mice (Fig. 1A, Supplementary 1B, Supplementary Tables 1, 2). Gastric organoids (GOs) derived from cells from the stomachs of young (7–8 week) wt or *Nfkb1*^*−/−*^ exhibited spheroid-like, cyst-like and budding morphologies (Fig. 1A, B, Supplementary 2) as previously described [9]. Quantitative bright field microscopy revealed that there were no significant differences between the total numbers of organoids, budding (total numbers) or budding potential (frequencies) between *Nfkb1*^*−/−*^ and control wt GOs on days 4, 6 or 8 of culture (Fig. 1C–F).

**Fig. 1:**
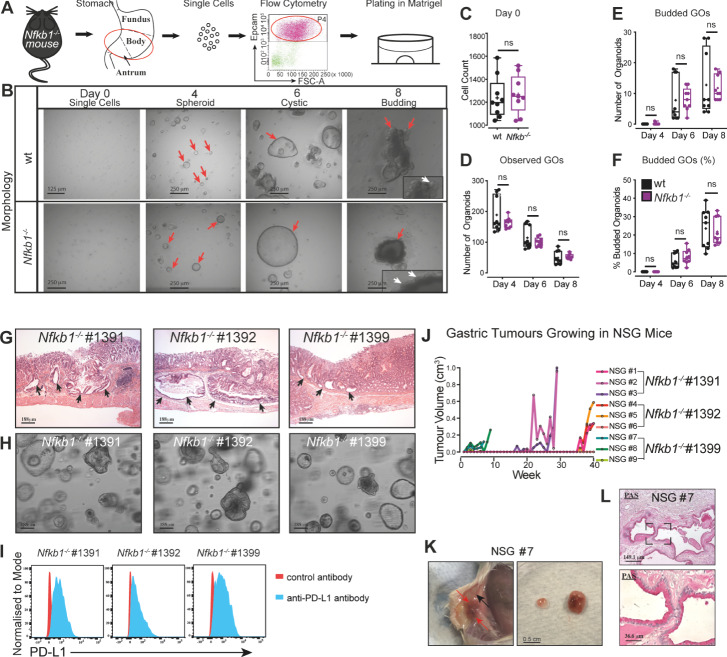
Establishment of gastroids of pre-neoplastic *Nfkb1*^*−/−*^ mice and PD-L1 expressing TGOs from GC of old sick *Nfkb1*^*−/−*^ mice. **A** Schematic for the culture of gastric epithelial cells. **B** Representative images of GO cultures showing morphologies (arrows). Graphical representation of **C** cell counts of sorted gastric epithelial cells, **D** GOs observed, inclusive of all spheroid, budding and cystic organoids **E** budding GOs and **F** budding GOs represented as a percentage of total observed GOs. **G** H&E stained sections of GCs from *Nfkb1*^*−/−*^ mice used to derive TGOs. Arrows indicate dysplasia or invasion. **H** Representative images of TGOs established from GC of *Nfkb1*^*−/−*^ mice. **I** Histogram showing PD-L1 expression on cells isolated from *Nfkb1*^*−/−*^ TGOs. **J** Tumor growth from subcutaneous injection of TGOs. Each line represents measurements from a single mouse. **K** Representative harvest of subcutaneous tumours derived from injected TGOs. Red arrows = tumour, black arrows = vascularisation. **L** Representative images of PAS staining (magenta) in transplanted TGOs. **C**–**F** Mean + /= SE (student’s *T*-test), *N* = 3 experimental repeats each with 3 replicates.

We also derived organoids from gastric tumours (TGOs) of *Nfkb1*^*−/−*^ mice. Gastric tumour tissue was harvested from these mice (>17 months, #1391, 1392, 1399) for histological examination, flow cytometric analysis and organoid culture. Histological examination of these mice confirmed dysplasia and gastric invasion of neoplastic cells (Fig. 1G). Consistent with our findings in pre-neoplastic *Nfkb1*^*−/−*^ mice [5, 6], which showed abnormally elevated PD-L1 protein expression on myeloid and epithelial cells in the stomach compared to wt control mice, flow cytometric analysis revealed high levels of PD-L1 on both CD11b^+^ myeloid cells and Epcam^+^ epithelial cells in the stomachs of all GC-burdened *Nfkb1*^*−/−*^ mice (Supplementary Fig. 3A). TGOs were generated from each tumour sample and expanded as described above (Fig. 1H, Supplementary Tables 1, 2, Supplementary methods). All TGOs exhibited either spheroid, cystic or budding morphology (Fig. 1H) and could be expanded for at least 30 passages (~60–100 days, Supplementary Fig. 3B). Following establishment of the method for the passaging of TGOs, a biobank of TGO lines from *Nfkb1*^*−/−*^ mice was initiated.

In all three independently derived *Nfkb1*^*−/−*^ TGOs (#1391, #1392 and #1399), PD-L1 expression remained high, even after repeated passaging and expansion in vitro (Fig. 1I). The TGOs were used for transplantation studies and injected subcutaneously into Nod-*scid*;common gamma chain^null^ (NSG) mice (Fig. 1J, Supplementary 3C). Within two months, tumour growth from TGO#1399 was observed in recipient NSG mice and from TGO#1391 and TGO#1392 at later time points (Fig. 1J, K). Macroscopic analysis revealed cystic tumour morphology (Supplementary Fig. 3C), reminiscent of the original tumours (Fig. 1G), and this was also confirmed histologically (Supplementary Fig. 3D). The transplanted TGOs stained positive for both neutral and acidic mucins, PAS and Alcian Blue (Fig. 1L, Supplementary 3D). Transplantation of *Nfkb1*^*−/−*^ TGOs was successful in 90% (8/9) of recipient NSG mice and was even observed, albeit with lower frequency (1/6), in young syngeneic *Nfkb1*^*−/−*^ recipient mice (Fig. 1J, Supplementary 3E). The reduced TGO uptake in syngeneic *Nfkb1*^*−/−*^ is likely due to the presence of a functional immune system (T and NK cells) not present in NSG mice.

In summary, GOs were generated from the stomachs of young healthy wt and young pre-neoplastic *Nfkb1*^*−/−*^ mice and TGOs from the GC of sick *Nfkb1*^*−/−*^ mice. The absence of NF-κB1 results in gastritis in mice with abnormally increased production of pro-inflammatory cytokines that drive inflammation leading to GC development [5, 6]. Cytokines can orchestrate a variety of responses in gastrointestinal stem cells, including proliferation and differentiation [10]. The GOs generated could therefore be used for assessing the impact of cytokines on the development of GC [5, 6] and to examine whether the loss of NF-κB1 accelerates the acquisition of oncogenic lesions. For instance, we have shown that *Nfkb1*^*−/−*^ mice that lack TNF have significantly reduced levels of PD-L1 in gastric epithelial cells compared to *Nfkb1*^*−/−*^ mice [6]. The GOs and TGOs can be expanded in vitro and frozen, providing a renewable bio-bank for genomic analysis, genetic manipulation, for example by using CRISPR/Cas9 technology [11]. They can also be used for testing novel therapies, particularly those aimed at killing cancer cells, since the TGOs retained expression of markers associated with evasion of immune attack that are relevant to certain human GC subtypes. Injection of TGOs into the stomach serosal layer may provide a more receptive niche for GC growth [12], including enhanced uptake in *Nfkb1*^*−/−*^ or even wt mice for the testing of therapeutic agents.

## Supplementary information

Supplementary Table 1

Supplementary Table 2

Supplemental Information

Supplementary Figure 1

Supplementary Figure 2

Supplementary Figure 3

## Data Availability

The data used to support the findings of this study are available from the corresponding author upon reasonable request.
