# Supplementary material for: Transplantable programmed death ligand 1 expressing gastroids from gastric cancer prone Nfkb1−/− mice
Source: Cell Death Dis. 2021 Nov 17;12(12):1091. doi: 10.1038/s41419-021-04376-2 (PMC8599488; doi:10.1038/s41419-021-04376-2)
Supplement: Supplementary file 1 — Supplementary Table 1 [file 41419_2021_4376_MOESM1_ESM.docx]

**Supplementary Table 1.** **Formulation and Reagent Source for Complete Medium Used for the Culturing of Gastric Organoids.**

| Reagents | Concentration | Source |
| --- | --- | --- |
| Advanced DMEM/F12 | - | Gibco Cat#12634010 |
| HEPES (1 M) | 10mM | Gibco Cat#15630080 |
| Penicillin-Streptomycin | 100 U/mL | Gibco Cat#15140122 |
| GlutaMAX (100x) | 1x | Gibco Cat#35050061 |
| B27 (50x) | 1x | Gibco Cat#17504044 |
| N2 (100x) | 1x | Gibco Cat#17502048 |
| Noggin | 10% | In-house conditioned medium |
| Wnt3a | 50% | In-house conditioned medium |
| R-spondin-1 | 20% | In-house conditioned medium |
| FGF10 | 100 ng/mL | Peprotech Cat#100-26B |
| EGF | 50 ng/mL | Peprotech Cat#31509 |
| Gastrin | 1 nM | Sigma Cat#G9145 |
| nAcetylcysteine (500 mM) | 1.25 mM | Sigma Cat#A9165 |
| Primocin | 100 μg/mL | Invivogen Cat#ANTPM1 |
| Rho-Kinase Inhibitor Y-27632 | 10 μM | Sigma Cat#Y0503 |

Medium Advanced DMEM/F-12, Dulbecco’s Modified Eagle Medium/Ham’s F-12.

Wnt-3a conditioned medium (WCM) was prepared using the cell line kindly provided by the Clevers laboratory (Hubrecht Institute, Netherlands). R-spondin-1 conditioned medium (RCM) was prepared using HEK293-HA-Rspo1-Fc cells, generated and kindly provided by the laboratory of Calvin Kuo (Stanford University). Noggin conditioned medium (NCM) was prepared using HEK293-mNoggin-Fc cells, generated and kindly provided by the Clevers laboratory (Hubrecht Institute, Netherlands).
