# Supplementary material for: Transplantable programmed death ligand 1 expressing gastroids from gastric cancer prone Nfkb1−/− mice
Source: Cell Death Dis. 2021 Nov 17;12(12):1091. doi: 10.1038/s41419-021-04376-2 (PMC8599488; doi:10.1038/s41419-021-04376-2)
Supplement: Supplementary file 2 — Supplementary Table 2 [file 41419_2021_4376_MOESM2_ESM.docx]

**Supplementary Table 2.** **Formulation and Reagent Source for Advanced DMEM/F12 Complete Medium.**

| Reagents | Concentration | Source |
| --- | --- | --- |
| Advanced DMEM/F12 | - | Thermo Cat#12634010 |
| HEPES (1 M) | 10mM | Thermo Cat#15630080 |
| Penicillin-Streptomycin | 100 U/mL | Thermo Cat#15140122 |
| GlutaMAX (100 x) | 1x | Thermo Cat#35050061 |
