# Supplementary material for: Transplantable programmed death ligand 1 expressing gastroids from gastric cancer prone Nfkb1−/− mice
Source: Cell Death Dis. 2021 Nov 17;12(12):1091. doi: 10.1038/s41419-021-04376-2 (PMC8599488; doi:10.1038/s41419-021-04376-2)
Supplement: Supplementary file 3 — Supplemental Information [file 41419_2021_4376_MOESM3_ESM.docx]

**Supplementary Information**

**METHODS**

**Mice**

All animal experiments complied with and were approved by The Walter and Eliza Hall Institute of Medical Research (WEHI) Animals Ethics Committee. *Nfkb1*^-/-^ mice ^1^ were originally generated on a mixed C57BL/6x129SV background using 129SV ES cells but had been backcrossed onto the C57BL/6 background for >10 generations prior to this study. *Nod-scid* common gamma chain^null^ (NSG) mice ^2^ carry the severe combined immune deficiency mutation and loss of common gamma chain, rendering them immunodeficient. Mice transplanted with GC-derived TGOs were 7-8 weeks of age. All animals were housed under conventional conditions in specific pathogen-free facilities and free of *Helicobacter* species.

**Gastric Gland Isolation, Cell Sorting and Tissue Culture**

Stomachs from 7 week-old wt and *Nfkb1^-/-^* mice were used to prepare GOs as outlined in Figure 1A, using an adaptation of previously published methods ^3^. Briefly, stomach body and antrum were harvested and digested in 2 mM EDTA/PBS with 0.5 mM DTT (Invitrogen Cat#P2325) for 15 min at 37°C, then shaken (x 30) to isolate epithelial cells. This process was repeated twice more. Fractions were combined and resuspended in Advanced DMEM/f12 Complete Medium (ACM, Supplementary Table 2) and passed through a 0.21 gauge needle (x 10) to obtain single cell suspensions. Enrichment for epithelial cells was performed by staining with APC-cy7 conjugated antibodies against EpCAM (G8.8, Biolegend #118218) and PI (1 μg/mL, Invitrogen #P3566), followed by sorting on a FACSARIA III (BD) to collect viable Epcam^+^/PI^-^ cells (Supplementary Figure 1). Epithelial cells were seeded at 2000 cells per drop (6 μL) in Matrigel Growth Factor Reduced (MGFR) (BD #356231) in a 24-well tissue culture plate (BD) pre-heated to 37°C. The MGFR was allowed to polymerise upside down for 15 min at 37°C and this was followed by the addition of 500 μL Complete Organoid Medium (Supplementary Table 1). Three 6 μL MGFR domes were seeded per well. Medium exchanges were performed every other day. Organoid cultures developed morphologies similar to those previously described ^4^.

**Organoid Live-Cell Imaging**

Imaging of Matrigel-embedded GOs was performed using a Zeiss Live-cell AxioObserver, under culture conditions (37°C/10% CO_2_) on days 0, 4, 6 and 8. Images were acquired automatically with stitch z-stacking, facilitating imaging of whole wells/domes. An extended depth of focus with contrast was applied to acquired images using ZEN 2 software.

**Organoid Counting**

Processed images were analyzed using Image J software. GOs were manually counted using the following criteria. Spheroids were defined as size of 18 μm – 150 μm. NGOs with extensions resembling gastric glands were counted as budding organoids. GOs with a cyst-like morphology were defined as >150 μm in size, with absence of budding.

**Histopathology**

Mouse stomachs were fixed in 10% formalin, paraffin embedded, and sections stained with haematoxylin and eosin (H&E), Periodic Acid-Schiff (PAS) or Alcian Blue (AB).

**Tumour Gastric Organoid (TGO) Preparation and Passaging**

Gastric tumour tissue was prepared from three *Nfkb1^-/-^* mice (>17 months of age), as described above for the GOs, and then digested using 0.6 mg/mL collagenase/dipsase (Roche Cat#11097113001) and 0.05% DNAse (Roche Cat#4716728001) as described previously ^5, 6^. Live isolated gastric cells were counted after staining with trypan blue, seeded in MGFR (20,000 live cells/30 µL MGFR) and cultured for 5-6 days in 500 μL Complete Organoid Medium (Supplementary Table 1).

To facilitate TGO passage, a method of isolating TGOs from MGFR was adapted from previously published methods ^7^ with the addition of collagenase/dispase digestion (Roche Cat#11097113001) 0.6 mg/mL to each well and incubated for 30 min at 37°C, then removed by aspiration. Ice-cold ACM (1 mL) was then added to each well and resuspended 5-6 times using a 1000 µL pipette tip with a 200 µL tip attached to disrupt the MGFR dome. This was followed by centrifugation at 1500 rpm for 5 min. The pellet was resuspended and trypsinized in 0.25% Trypsin/ACM for 5 min at 37°C. Following trypsinization, the organoids were centrifuged and the pellet washed with balanced salt solution (BSS)/10% FCS and resuspended in ACM. Cells were pelleted by centrifugation and resuspended in MGFR on ice. MGFR was seeded per well (30 μL) of a 24-well tissue culture place and allowed to polymerize for 15 min, followed by the addition of 500 μL Complete Organoid Medium (Supplementary Table 1). TGOs were passaged every 3-4 days and could be stored in liquid N2 (10% DMSO/90% ACM).

**Flow Cytometric Analysis of PD-L1 Expression**

Single cell suspensions were stained with APC-cy7-conjugated antibodies against EpCAM (Biolegend #118218), BV605-conjugated antibodies against CD11b (BD Biosciences #563015), BV421-conjugated Ig-isotype control antibody (rat IgG2bk; Biolegend #400639) or BV421-conjugated antibodies against PD-L1 (Biolegend #124315), with PI (1 μg/mL) (Invitrogen #P3566). Samples were analysed on an LSR IIC (Becton Dickinson) or Fortessa 1 (Becton Dickinson). For analysis, raw data were normalised to mode on Flowjo v10.

**Immunohistochemistry**

Tissue sections were de-paraffinized according to standard protocols ^5,6^. For epitope retrieval, tissue sections were boiled in citrate buffer (10 mM Na citrate, pH 6.0) for 2 x 5 min. Slides were incubated overnight at 4^o^C with an antibody against p STAT1 (phospho S727, abcam109461). Ab detection was performed using biotinylated goat anti-rabbit IgG antibodies (Vector Laboratories# BA-9400, # BA-1000) followed by incubation with the ABC Elite detection kit (Vector Laboratories# PK-6100) with diaminobenzidine as the substrate. All photomicrographs were acquired using a 5NA 0.15 or 10 NA 0.3 objective lens attached to an Axioplan 2 (Carl Zeiss) microscope.

**Subcutaneous Transplantation of TGOs**

Prior to subcutaneous injection, GC-derived TGOs in culture were isolated from Matrigel by collagenase/dispase digestion and fragmented by mechanical disruption as described above. Briefly, after collagenase/dispase digestion, ice-cold ACM (1 mL) was added to each well and resuspended 6-7 times to dissociate the MGFR domes and fragment TGOs. TGOs were then transferred into an Eppendorf tube, pelleted by centrifugation (1500 rpm) and resuspended in 50 μL of MGFR at 4°C for each transplant. An equal volume of Complete Organoid Medium was added to the MGFR/TGO suspension and the 100 μL of suspension was aspirated into a 1 mL syringe with a 21G needle. Syringes including the needles were chilled prior to subcutaneous injection into the flank of recipient mice. Each recipient mouse was injected with a minimum of 12 wells worth of MGFR domes embedded with TGOs in a volume of 30 μL. Palpable tumours were measured using high-precision calipers (Vernier) for calculation of tumour volumes twice weekly.

**Statistical Analysis**

All statistical analyses were performed using GraphPad Prism 7 software. Comparisons between mean values were performed using a two-tailed student’s t-test. p values greater than 0.05 were considered non-significant. Error bars represent SEM.

**References For Supplementary Material**

1. Sha WC, Liou H-C, Tuomanen EI, Baltimore D. Targeted disruption of the p50 subunit of NF-kB leads to multifocal defects in immune responses. *Cell* **80**, 321-30 (1995)­.

2. Shultz LD, Goodwin N, Ishikawa F, Hosur V, Lyons BL, Greiner DL. Human cancer growth and therapy in immunodeficient mouse models. *Cold Spring Harb Protoc*. **7**, 694-708 (2014).

3. Sato T, Vries RG, Snippert HJ, van de Wetering M, Barker N, Stange DE, et al. Single Lgr5 stem cells build crypt-villus structures in vitro without a mesenchymal niche. *Nature*  **459 7244**) 262-5 (2009).

4. Barker N, Huch M, Kujala P, van de Wetering M, Snippert HJ, van Es JH, et al. Lgr5(+ve) stem cells drive self-renewal in the stomach and build long-lived gastric units in vitro. *Cell Stem Cell* **6**(1), 25-36 (2010).

5. O'Reilly LA, Putoczki TL, Mielke LA, Low JT, Lin A, Preaudet A, et al. Loss of NF-kappaB1 Causes Gastric Cancer with Aberrant Inflammation and Expression of Immune Checkpoint Regulators in a STAT-1-Dependent Manner. *Immunity* 2018; **48**(3), 570-83 e8 (2018).

6. Low JT, Christie M, Ernst M, Dumoutier L, Preaudet A, Ni Y, et al. Loss of NFKB1 Results in Expression of Tumor Necrosis Factor and Activation of Signal Transducer and Activator of Transcription 1 to Promote Gastric Tumorigenesis in Mice. *Gastroenterology* **159**(4), 1444-58 (2020).

7. Mahe MM, Aihara E, Schumacher MA, Zavros Y, Montrose MH, Helmrath MA, et al. Establishment of Gastrointestinal Epithelial Organoids. *Curr Protoc Mouse Biol.* **3**(4), 217-40 (2013).

**Supplementary Figure Legends**

**Supplemental Figure 1. Stomach Immunohistochemistry and Gating Strategy for Flow Cytometric Cell Sorting. (A)** Representative staining for phospho-STAT1 in the gastric antrum of 3-4 months old wt and *Nfkb1*^-/-^ mice (n=4/genotype). Arrowheads indicate positive staining (brown), scale bar represents 96 μm. **(B)** Representative flow chart showing flow cytometry sort gates for collecting live Epcam^+^ PI^-^ gastric cells from which GO or TGOs were derived.

**Supplemental Figure 2. Establishment of Gastric Organoids from Young, Healthy wt and *Nfkb1^-/-^* mice.** Representative images of GO cultures that had been derived from single-sorted epithelial cells from the stomachs of young (3 month) healthy wt or *Nfkb1^-/-^* mice and their observed morphologies over a period of 9 days. Matrigel domes (6 μL) were imaged on days 0, 4, 6 and 8 and images stitched together using Zen software (Zeiss). N=3 experiments per genotype with 3 replicates per experiment.

**Supplemental Figure 3. Characteristics of Gastric Tumours from *Nfkb1^-/-^* Mice, Establishment of Tumour Gastric Organoids (TGOs) from Gastric Cancers of Aged, Sick *Nfkb1^-/-^* Mice** **and *in vivo* Transplantation of these TGOs.** (A) Flow cytometric analysis of PD-L1 expression on gastric epithelial (Epcam^+^) cells and myeloid (CD11b^+^) cells isolated from each of the *Nfkb1^-/-^* gastric tumours used to establish the TGO lines. Cells isolated from each tumour tissue sample were stained with either an Ig isotype-matched control antibody or an antibody against PD-L1. The histograms depict raw data, normalised to mode using Flowjo v10 software. (B) Representative images of TGO lines derived from the GC of *Nfkb1^-/-^* mice (N=3), during the early days of establishment and at extended passage. (C) Photomicrographs showing injection site (red arrow) and harvest of subcutaneous cystic tumours from NSG #7 and NSG #8 mice that had been injected with TGO *Nfkb1^-/-^* #1399 (red arrows indicate cystic tumour lesions and black arrows indicate vascularisation). (D) Representative H&E, PAS and AB stained sections of cystic tumours harvested from NSG #7 and NSG #8 mice that had been injected with TGOs derived from GC of aged, sick *Nfkb1^-/-^* mice, at harvest. PAS staining (magenta) indicates the presence of neutral mucins. AB staining (blue) indicates the presence of acidic mucins. (E) Weekly growth of tumours (cm^3^) generated from subcutaneous injection of TGOs of the indicated lines into the flanks of young (7 week) *Nfkb1^-/-^* mice. Each line represents tumour measurements from a single mouse.

**Supplementary Table 1.** Formulation and Reagent Source for Complete Medium Used for the Culturing of Gastric Organoids.

**Supplementary Table 2.** Formulation and Reagent Source for Advanced DMEM/F12 Complete Medium.
