# Supplementary figures and images for: Transplantable programmed death ligand 1 expressing gastroids from gastric cancer prone Nfkb1−/− mice
Source: Cell Death Dis. 2021 Nov 17;12(12):1091. doi: 10.1038/s41419-021-04376-2 (PMC8599488; doi:10.1038/s41419-021-04376-2)

Supplementary Figure 1

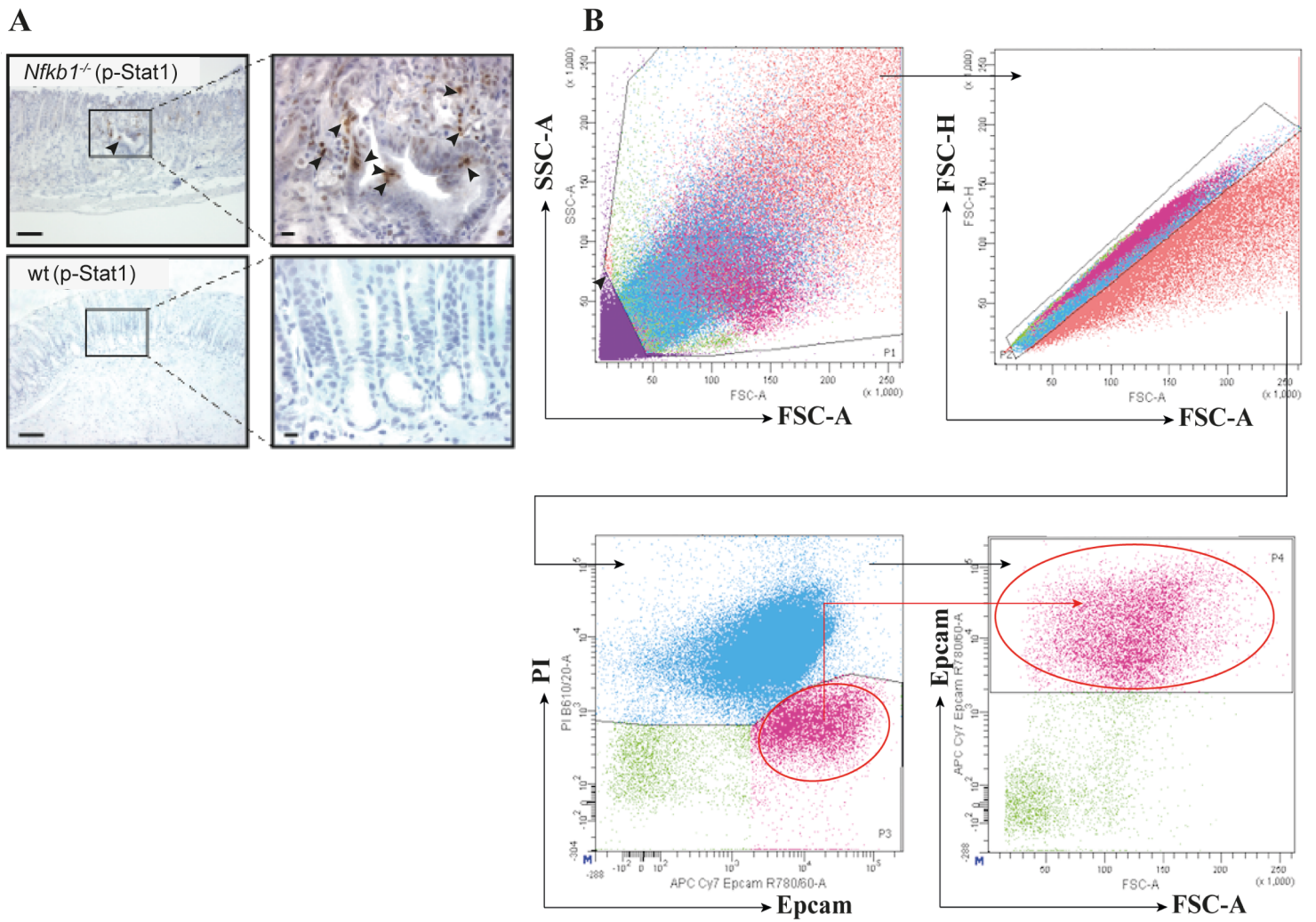

Supplement: Supplementary file 4 — Supplementary Figure 1 [file 41419_2021_4376_MOESM4_ESM.pdf]

Supplementary Figure 2

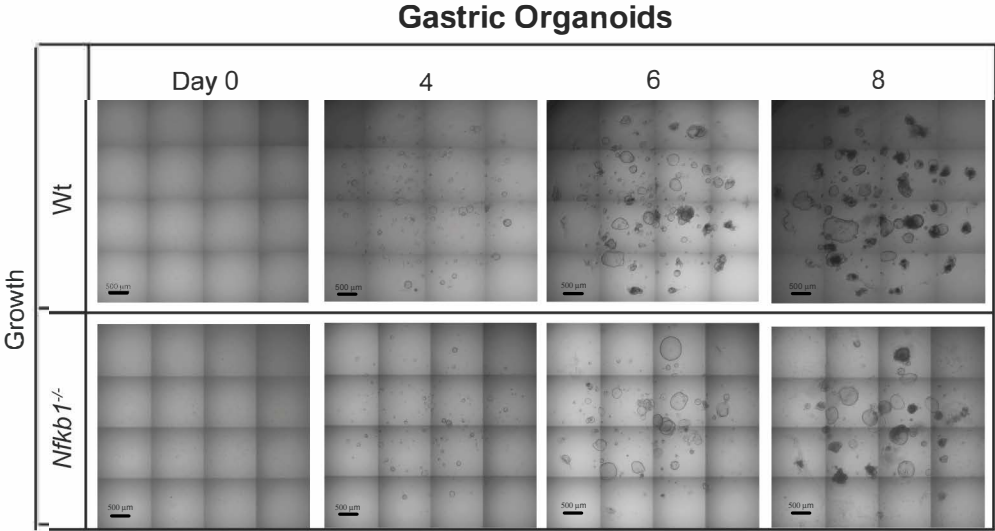

Supplement: Supplementary file 5 — Supplementary Figure 2 [file 41419_2021_4376_MOESM5_ESM.pdf]

## Supplementary Figure 3

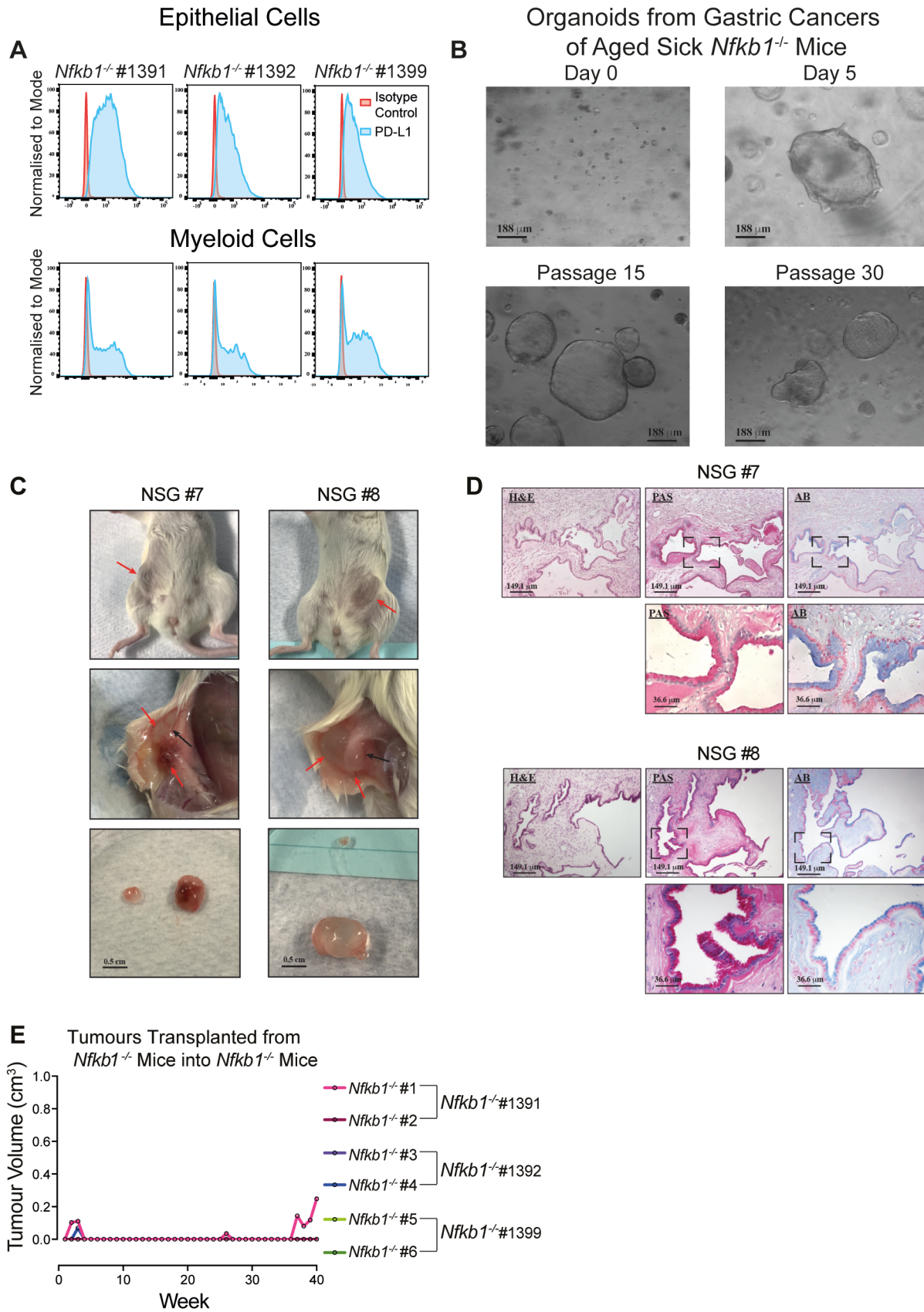

Supplement: Supplementary file 6 — Supplementary Figure 3 [file 41419_2021_4376_MOESM6_ESM.pdf]
